# Validation of a semi-quantitative scoring system and workflow for analysis of fluorescence quantification in companion animals

**DOI:** 10.3389/fvets.2024.1392504

**Published:** 2024-07-31

**Authors:** Ann S. Ram, Kathy Matuszewska, Charly McKenna, Jim Petrik, Michelle L. Oblak

**Affiliations:** ^1^Department of Biomedical Sciences, University of Guelph, Guelph, ON, Canada; ^2^Department of Clinical Studies, University of Guelph, Guelph, ON, Canada

**Keywords:** indocyanine green, fluorescence-guided surgery, NIR imaging, canine cancer, sentinel lymph node (SLN) mapping

## Abstract

**Significance:**

Many commercially available near-infrared (NIR) fluorescence imaging systems lack algorithms for real-time quantifiable fluorescence data. Creation of a workflow for clinical assessment and *post hoc* analysis may provide clinical researchers with a method for intraoperative fluorescence quantification to improve objective outcome measures.

**Aim:**

Scoring systems and verified image analysis are employed to determine the amount and intensity of fluorescence within surgical specimens both intra and postoperatively.

**Approach:**

Lymph nodes from canine cancer patients were obtained during lymph node extirpation following peritumoral injection of indocyanine green (ICG). First, a semi-quantitative assessment of surface fluorescence was evaluated. Images obtained with a NIR exoscope were analysed to determine fluorescence thresholds and measure fluorescence amount and intensity.

**Results:**

*Post hoc* fluorescence quantification (threshold of Hue = 165–180, Intensity = 30–255) displayed strong agreement with semi-quantitative scoring (*k* = 0.9734, *p* < 0.0001). Fluorescence intensity with either threshold of 35–255 or 45–255 were significant predictors of fluorescence and had high sensitivity and specificity (*p* < 0.05). Fluorescence intensity and quantification had a strong association (*p* < 0.001).

**Conclusion:**

The validation of the semi-quantitative scoring system by image analysis provides a method for objective *in situ* observation of tissue fluorescence. The utilization of thresholding for ICG fluorescence intensity allows *post hoc* quantification of fluorescence when not built into the imaging system.

## 1 Introduction

Indocyanine green (ICG) is a frequently reported fluorescent agent utilized for intraoperative sentinel lymph node (SLN) mapping and tumor bed imaging (1–3) due to its high signal-to-background ratio (SBR), low autofluorescence in tissue, nontoxicity, and accessibility (4, 5). With an excitation peak of 780 nm and an emission peak of 820 nm, ICG is a fluorescent near-infrared (NIR) tracer within the NIR spectrum (4–6). This tracer is non-specific and, when injected intravenously, binds readily to intravascular plasma proteins (4, 7), whereas peritumorally injected ICG will interact with proteins in the lymph (8). Near-infrared fluorescence imaging with ICG provides improved resolution in SLN mapping procedures compared to radioisotopes, blue dyes or the combination of radiocolloids and visible dyes traditionally used in veterinary medicine (4, 9). While ICG is commonly used in human medicine, ICG-NIR is viewed as an emerging technology in veterinary medicine, gaining traction in companion animal patients for oncologic surgeries as well as many other applications.

Various NIR imaging systems are used in tandem with ICG, each resulting in differing NIR light depth of penetration (3, 4, 10–16). A limited number of clinically approved imaging systems have built-in fluorescence quantification algorithms and processing with arbitrary units, including SPY Elite (Novadaq Technologies, Waterloo, Canada), EleVision IR (Medtronic, Dublin, Ireland) and IC-View (Pulsion Medical Systems AG, Munich, Germany) (17–20). Other approved open-surgery imaging systems including Photodynamic Eye or PDE Neo (PDE, Hamamatsu Photonics K. K., Honshu, Japan), FLARE (Curadel, Massachusetts, United States), Fluobeam (Fluoptics, Grenoble, France), and VITOM II ICG (Karl Storz, Tuttlingen, Germany); do not have the ability to quantify fluorescence *in situ* or *ex vivo* (17). The challenge in fluorescence-guided surgery is the lack of built-in fluorescence quantification or intensity algorithms as this limits the ability to objectively compare fluorescence intensity between procedures and therefore hinders a researcher’s ability to efficiently compare surgical techniques, accurately assess fluorophores and develop protocols for clinical research and practice (21). Therefore, there is a need to provide imaging procedures with standardized methods for the quantification of fluorescence in order to advance future research (21–23). Furthermore, researchers and clinicians lack methods for real-time quantification of fluorescence and resort to subjective reporting of the presence or absence of fluorescence (24–28) which impairs the ability to make strong scientific conclusions (21). Image analysis protocols for fluorescence are also catered towards fixed tissues and cells that utilize standard immunofluorescence and microscopy (29, 30). Currently, reporting standards for fluorescence-guided surgical specimens recommend SBR to quantify fluorescence (21), however, protocols vary depending on imaging system, tracer and specimen type. Additionally, with the commonality of equipment in human and veterinary medicine and the similarity of naturally occurring diseases between species, there is cross-over potential in applying these analysis methods. Therefore, it is important to provide defined protocols when assessing fluorescence to limit bias and increase reproducibility (21).

This study aims to provide a simple, validated intraoperative semi-quantitative method to evaluate fluorescence. We hypothesize that a scoring system would benefit researchers and clinicians when built-in quantification algorithms or post-hoc analysis are unavailable. This study also depicts optimized parameters within image analysis to assess fluorescence amount and intensity in lymph nodes. We postulate that this will assist in the standardization of the analysis process when evaluating whole tissue specimens.

## 2 Materials and methods

Lymph nodes were obtained prospectively from 17 canine patients with naturally occurring cancer enrolled in a study involving complete regional lymph node bed extirpation for comparison with ICG SLN mapping techniques at the Ontario Veterinary College Health Sciences Centre from 2018 to 2019 (AUP 3606-008R). For each patient, pharmaceutical grade ICG (IC-Green, NDC 17478–701-02, Akorn, Illinois, United States) was reconstituted according to label instructions. All dogs had a 1 mL (0.25 mg/mL) ICG solution injected peritumorally. All lymph nodes in the basin, positive or negative for fluorescence, were removed during routine regional lymph node extirpation.

### 2.1 Proposal of a semi-quantitative scoring system for lymph node imaging

The VITOM II ICG system (λ_excitation_ = 805 nm, λ_emission_ = 835 nm) consists of a telescope connected to a SPIES 3-chip high-definition IMAGE1 S camera head with Karl Storz NIR/ICG camera (27, 28). Using the VITOM II ICG system, fluorescence appears as blue on the monitor. The imaging system was fixed above a table with an articulating stand positioned perpendicular focal distance of 20 cm from the specimen. Fluorescence scores were assigned as follows: 0 = no fluorescence, 1+ = 1–25% of the node surface fluorescent, 2+ = 26–50% fluorescent, 3+ = 51–75% fluorescent, and 4+ = 76–100% fluorescent. Lymph nodes were imaged in a standardized manner using a white background and consistent height parameters (26). Images from the VITOM II ICG were formatted with pseudo-colored blue fluorescence and raw images (grayscale) were not available from this system. Scoring was performed intraoperatively and 1-h after lymph node extirpation. The surgeon (MO) determined visual fluorescence status (positive or negative) and assigned a score immediately following removal of the lymph node. Within 1 h of extirpation, all lymph nodes, whether positive or negative, were assessed by a blinded investigator (SR) and assigned an *ex vivo* fluorescence score using the same scoring system. At the same time as scoring (28), still fluorescent images were obtained of both sides of each lymph node and saved for future assessment. The postoperative *ex vivo* score was given to both sides of the lymph node by a blinded investigator and the scored side with the clearest visible fluorescence and minimum fat tissue was used for analysis.

### 2.2 Validation of semi-quantitative scoring system

Intraoperative scores and blinded postoperative scores were evaluated for further analysis of agreement at least 4 weeks following surgery to assess scoring based on *ex vivo* images. Images of each lymph node were assigned a number and randomized for evaluation and image analysis. Each image was assigned into 4 groups of 21, consisting of 83 images analyzed in total, which was done with a random set generator (GraphPad Randomizer, California, United States). One of the groups has only 20 images due to the uneven number of total images analyzed. Two investigators, blinded to the original findings, then assessed the amount of surface fluorescence in each image and assigned a score as described above.

### 2.3 Verification of image analysis method

The image analysis process was verified with positive and negative controls. Negative controls (*n* = 5) were lymph nodes with no exposure to ICG, previously collected from canine patients. Positive controls (*n* = 18) were lymph nodes that scored 4+ on the semiquantitative scale. These controls were used to set the hue and intensity histogram in an image analysis software (MetaMorph^®^) (31). The set of lymph nodes used as positive controls for fluorescence was based on the totality of the fluorescence signal on the surface of the lymph node and brightness depicting unambiguous results. Using principles from fluorescence microscopy analysis, rigor and reproducibility were introduced into the analysis process (32). Controls for autofluorescence were accounted for by using clean white backgrounds and thresholding strictly (31, 32). To avoid introducing bias and subjectivity, saturation was not adjusted for the intensity and surface fluorescence analysis. Surface area of fluorescence was modeled after SBR where fluorescence signal is divided against background signal (32, 33). Signal acquisition was validated by color thresholding that distinguished the fluorescent area from the non-fluorescent areas of the lymph node. Signals outside the specified threshold were excluded from the analysis. Average fluorescence intensity (FI), the amount of light emitted, was automatically calculated by the image analysis software based on set threshold parameters. Typically, FI measures account for background noise; however, the standardized imaging conditions prevented any background signal. This was validated in image analysis where background FI was consistently 0 across all images and compared to max intensity (33).

### 2.4 Quantification of surface fluorescence signal using image analysis

Randomized images of the clearest visible fluorescence and minimum fat tissue were processed and analyzed in MetaMorph (Molecular Devices, California, United States). Images were cropped and a Median Filter was applied. A Region of Interest (ROI) was drawn manually around the whole lymph node. Using the “Color Threshold” function, two threshold settings (A and B) were applied to every image. The hue and intensity of pixels were measured with a range of 0 to 255. Threshold A performed analysis using the following settings: Hue = 165–180 and Intensity = 30–255. Threshold B performed analysis using the following settings: Hue = 165–180 and Intensity = 45–255. Saturation was not altered in any of the images. The area of the lymph node ROI and the area of the detected signal in the measure of pixels were generated by the program. A percentage of the threshold signal in the ROI was used to measure the amount of fluorescence detected and calculated automatically by the software depicted by Eq. (1).


(1) $$\mathrm{Amount}\;\mathrm{of}\;\mathrm{surface}\;\mathrm{fluorescence}=\frac{\begin{array}{l} \mathrm{area}\;\mathrm{of}\;\mathrm{signal}\;\\ \mathrm{detected}\; \end{array}}{\begin{array}{l} \mathrm{area}\;\mathrm{of}\;\mathrm{lymph}\;\\ \mathrm{node} \end{array}}\times 100$$

### 2.5 Quantification of fluorescence intensity using image analysis

Images the clearest visible fluorescence and minimum fat tissue were analyzed in tandem with the previous analysis (2.4). As previously described, a ROI of the surface image of one side of the lymph node was generated. For the background ROI, a uniform circle near the lymph node was used. The same circle (area = 5,806 pixels) was used for all images. The “Color Threshold” function was used and three threshold settings (1–3) were applied for intensity. Hue was kept consistent for all three threshold settings at 165–180. Threshold 1 intensity was 30–255, Threshold 2 intensity was 35–255, and Threshold 3 intensity was 45–255. Since 24-bit color images were analyzed, the output was configured to a “Blue” color channel and “threshold for intensity measurements” was selected to only include the gray value intensities within the specified threshold range. For each ROI, the average FI was obtained as a grayscale value (arbitrary units, AU) and calculated by the software using Eq. (2). Intensity measurement terms are used to describe and quantify grayscale intensity (pixel brightness) values.


(2) $$\begin{array}{l} \mathrm{Average}\;\mathrm{fluorescence}\;\mathrm{intensity}=\left(\frac{\sum \mathrm{Gray}\;\mathrm{value}}{\mathrm{Number}\;\mathrm{of}\;\mathrm{pixels}}\right)-\\ \mathrm{Backgroundsignal}\; \end{array}$$

### 2.6 Statistical analysis

Statistical analyses were completed using commercially available software SAS 9.4 (SAS Institute Inc., Cary, NC) by a biostatistician (G.M.) who was blinded to the specimen collection and assessment process. The percentage of fluorescence on the surface of the lymph nodes was not normally distributed, therefore a non-parametric Wilcoxan-Mann–Whitney was used to compare the differences between the values from Threshold A and Threshold B. Cohen’s weighted kappa and simple kappa (k) statistics were used to assess the concordance of semi-quantitative scoring, surface fluorescence analysis, fluorescence status, and inter- and intraobserver agreement. Weighted kappa and simple kappa coefficients with 95% confidence intervals (CI) were calculated to determine the strength of agreement. Kappa coefficients that fell within 0.21–0.40 were interpreted as fair agreement, 0.41–0.60 as moderate agreement, 0.61–0.80 as substantial agreement, and 0.81–1 as almost perfect agreement (34). A non-parametric Friedman test was applied to compare the average intensity values for Threshold 1, 2, and 3. Logistic regressions were used to generate the empirical receiver operating curve (ROC) of sensitivity and 1-specificity for all three FI thresholds in the data set. The ROC provided an optimal predicted probability cut-off for these intensity thresholds. The area under the ROC curve (AUC) is the average sensitivity of the thresholds over the range of specificities to represent the overall performance of the continuous threshold variable. Spearman rank-order correlation was used to measure the strength of monotonic relationship between average FI and surface amount of fluorescence. The r^2^ value in this test indicates the strength of a relationship between two sets of data; as r^2^ values become closer to 1, the stronger the relationship. Statistical significance was set at a two-sided *p*-value less than 0.05.

## 3 Results

**Figure 1:**
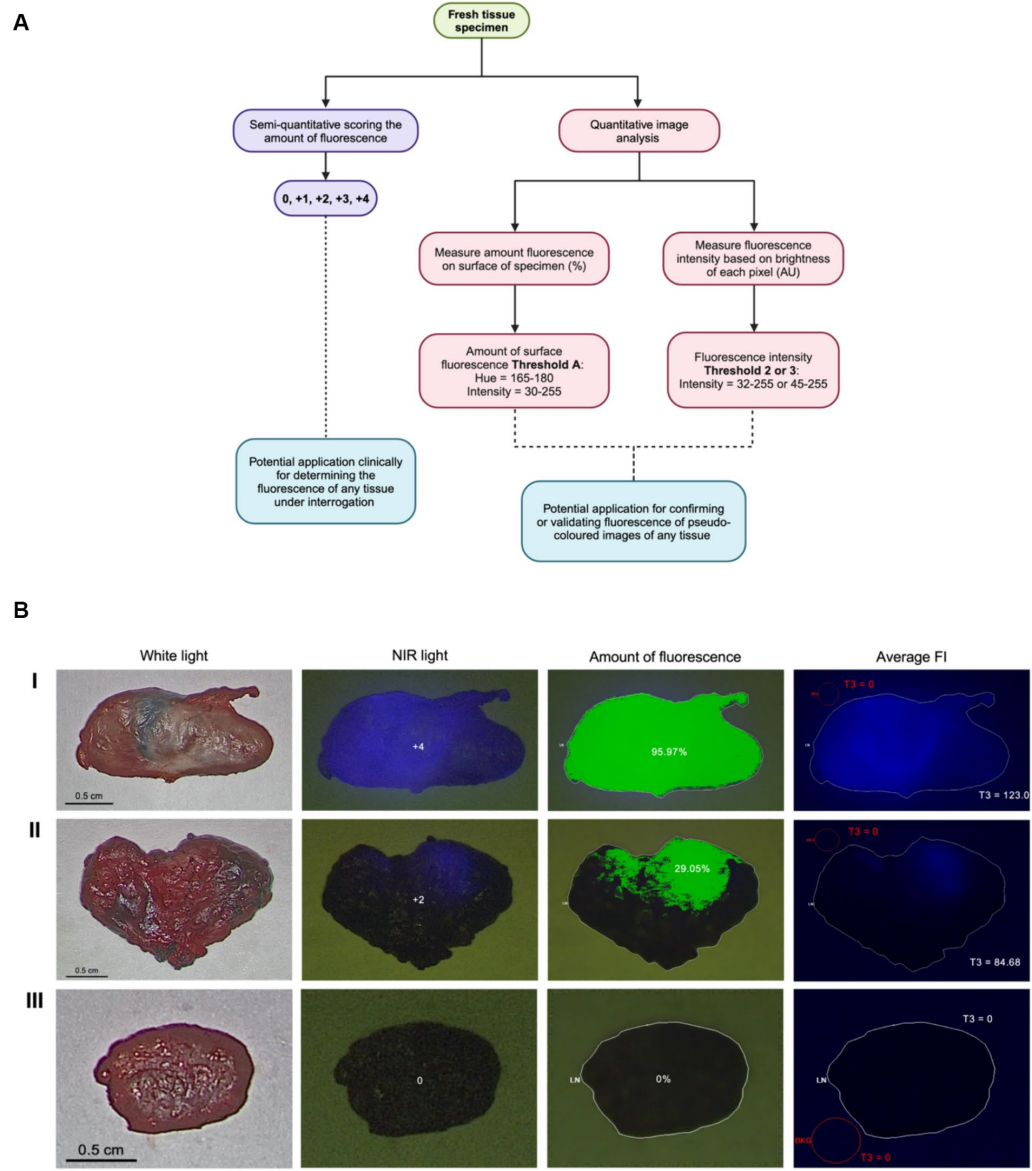
Visual assessment and image analysis workflow and outcomes. **(A)** Workflow for quantifying ICG fluorescence semi-quantitatively and through image analysis. **(B)** Imaging methods, score and analysis of highly fluorescent node (I), slightly fluorescent node (II) and true negative node (III) using Threshold A for the amount of fluorescence and Threshold 3 (T3) for FI.

A total of 87 lymph nodes were collected from 17 dogs with naturally occurring cancer and included in the analysis (Supplementary Table S1). Four specimens were excluded due to poor image quality or the small size of the lymph node, resulting in 83 standardized images with complete scoring and analysis using Threshold A for the amount of surface fluorescence and both Threshold 2 and 3 for fluorescence intensity (Figure 1A). Threshold B for the amount of surface fluorescence and Threshold 1 for fluorescence were omitted from the workflow due to poor significance and agreement. Most lymph nodes (58%; 48/83) were identified *in situ* as fluorescence positive, falling into 1+ (23%; 11/48), 2+ (10%; 5/48), 3+ (29%; 14/48), and 4+ (38%; 18/48) scoring categories. The remainder of the lymph nodes (42%; 35/83) were fluorescence negative. Image analysis was successfully performed for all lymph node images using MetaMorph for intensity and surface fluorescence (Figure 1B). Analysis of negative controls picked up 0% surface amount of fluorescence and FI of 0 AU (Supplementary Table S2).

The mean difference of agreement between Threshold A and Threshold B for surface fluorescence analysis was statistically significant (*p* = <0.0001). Threshold B, in comparison, had lower agreement values in all tested settings and types of scoring (Supplementary Table S3A), resulting in the disuse of this analysis parameter to quantify the amount of fluorescence. There was an almost perfect concordance between *ex vivo* scores and fluorescence status (positive or negative) against analysis using Threshold A (*k* = 0.9734 [0.95–1], *p* < 0.0001 and *k* = 0.9752 [0.93–1], *p* < 0.0001, respectively) (Table 1). A strong intraobserver and interobserver agreement was found for visual scores designated by the two investigators (*k* = 0.9732 [0.95–1], *p* < 0.0001 and *k* = 0.8900 [0.84–0.94], *p* < 0.0001, respectively) and almost perfect agreement in assessing fluorescence status between (*k* = 0.9267 [0.85–1], *p* < 0.0001). When comparing different investigator lymph node fluorescence scores to image analysis, there was an excellent agreement for Threshold A analysis (Table 1). Lastly, there was perfect agreement between scores obtained intraoperatively and postoperatively (*k* = 0.9267 [0.88–0.97], *p* < 0.000). In addition, when Threshold A was compared against intraoperative scoring, Threshold A had excellent agreement (Table 1).

**Table 1:** Agreement values and kappa coefficients comparing scoring, analysis and scoring settings.

	Percentage agreement	Weighted kappa (95% CI)	Two-sided *p*-value
Score vs. Threshold A	95.2%	0.9734 (0.95–0.1)	<0.0001
Visual fluorescence status vs. Threshold A	98.8%	0.9752 (0.93–1)	<0.0001
Intraobserver	95.2%	0.9732 (0.95–0.1)	<0.0001
Interobserver	80.7%	0.8900 (0.84–0.94)	<0.0001
Observer 1 vs. Threshold A	90.4%	0.9469 (0.91–0.98)	<0.0001
Observer 2 vs. Threshold A	73.5%	0.8508 (0.80–0.90)	<0.0001
Intraoperative vs. postoperative	87.9%	0.9254 (0.88–0.97)	<0.0001
Intraoperative vs. Threshold A	83.1%	0.8994 (0.85–0.95)	<0.0001

**Figure 2:**
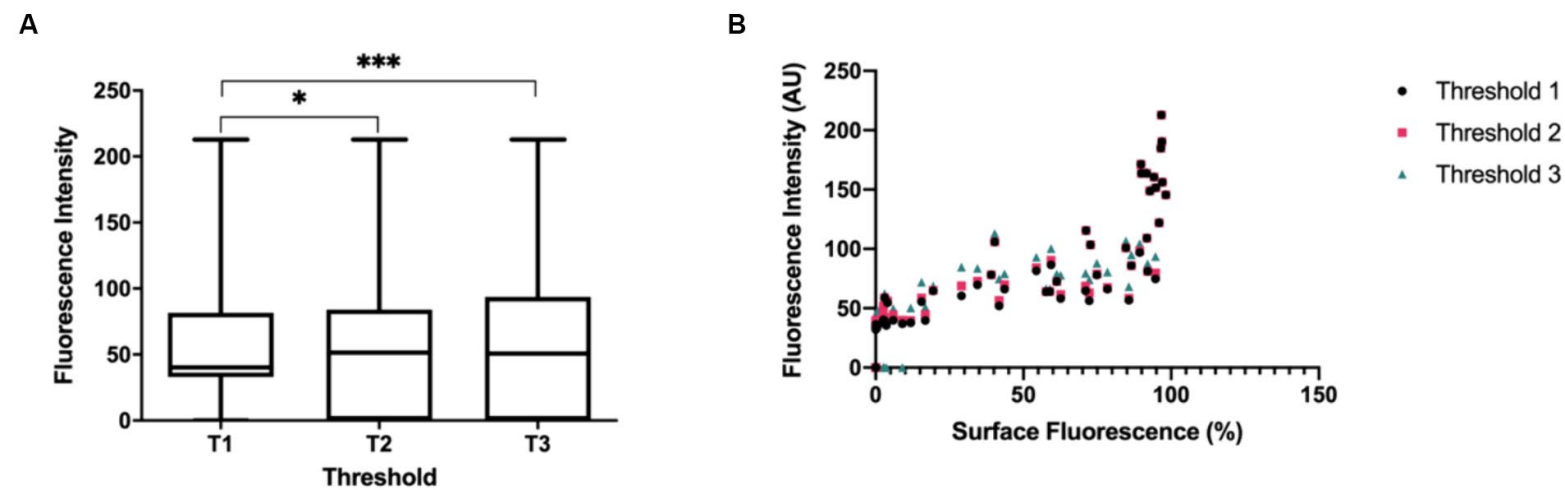
**(A)** Boxplots depicting the comparison of average FI (output measurement of the image analysis program) between Threshold 1 (T1), Threshold 2 (T2) and Threshold 3 (T3). Average FI between T1 (*S* = 143.5, *Mdn* = 40.3) vs. T2 (*S* = 172.5, *Mdn* = 51.6), T1 vs. T3 (*S* = 182.0, *Mdn* = 50.8), and T2 vs. T3. Asterisk denotes statistical significance. *N* = 83 **(B)** Relationship between average FI and surface fluorescence amount (Threshold A) on lymph node specimens. The boxplots and statistical tests report the medians of the output measurements collectively.

For each lymph node image, the average FI was assessed (Figure 2A). The average FI between Thresholds 1 and 2 and Thresholds 1 and 3 were significantly different (*Z* = −2.79701, *p* = 0.0052) and (*Z* = −3.71327, *p* = 0.0002), while the average FI between Thresholds 2 and 3 was not significantly different (*Z* = −0.91626, *p* = 0.3595).

Logistic regression demonstrated that the average FI values could predict the probability of negative or positive status for fluorescence from visual scoring and gold standard (fluorescence amount analysis) with significance for all intensity thresholds compared to dichotomous visual status. This resulted in all tested threshold settings being good predictors of nodal fluorescence for positive or negative status (Table 2). Specifically, for every Threshold 1 average FI gray unit increase, there was a 2.486 (CI 1.279–4.831) chance of the node being fluorescent based on visual assessment. The Threshold 1 setting produced an 87.6% sensitivity and 97.1% specificity with an intensity cut-point value of 39.95. For every Threshold 2 average FI unit increase there was 1.415 (CI 1.005–2.553) odds of the node being fluorescent. Threshold 2 modelled a sensitivity of 91.7% and specificity of 97.1% with an intensity cut-point of 44.56. For every Threshold 3 average FI unit increase there was a 1.073 (CI 1.042–1.105) chance of the node being fluorescent, therefore Threshold 3 resulted in an 89.6% sensitivity and 94.3% specificity with an intensity cut-point of 48. However, for every FI unit increase using Threshold 3, there was a 1.085 (CI 2.042–1.129) chance of the lymph node being fluorescent in the gold standard assessment (Table 3). This modeled an 89.8% sensitivity and 97.1% specificity with a cut-point of 48 (Table 4). Lastly, there was an almost perfect association between average FI values of all threshold settings and amount of fluorescence from Threshold A due to coefficients (Table 5).

**Table 2:** Analysis of maximum likelihood and odds ratio estimates for FI predicting visual negative or positive fluorescence status.

	Maximum likelihood estimate					Odds ratio estimates	
Intensity setting	df	Estimate	*s*	Wald c^2^	Pr > c^2^	Point estimates	Wald 95% CI limits
Threshold 1	1	0.9106	0.3390	7.2151	0.0072	2.486	1.279–4.831
Threshold 2	1	0.4712	0.2378	3.9282	0.0475	1.602	1.005–2.553
Threshold 3	1	0.0702	0.0150	21.8892	<0.0001	1.073	1.042–1.105

s, standard deviation.

**Table 3:** Analysis of maximum likelihood and odds ratio estimates for FI predicting gold standard negative or positive fluorescence status.

	Maximum likelihood estimate					Odds ratio estimates	
Intensity setting	df	Estimate	*s*	Wald c^2^	Pr > c^2^	Point estimates	Wald 95% CI limits
Threshold 1	1	2.0849	1.1691	3.1802	0.0745	-	-
Threshold 2	1	1.4555	0.8698	2.8000	0.0943	-	-
Threshold 3	1	0.0813	0.0203	16.0793	<0.0001	1.085	1.042–1.129

s, standard deviation; -, not reported due insignificant *p*-value.

**Table 4:** Classification table for predictors of fluorescence status.

Intensity setting	Sensitivity	Specificity	Cut-point
Visual fluorescence positive/negative status			
Threshold 1	87.6	97.1	39.95
Threshold 2	91.7	97.1	44.56
Threshold 3	89.6	94.3	48
Gold standard fluorescence positive/negative status			
Threshold 3	89.8	97.1	48

**Table 5:** Correlation coefficients and r-squared variance based on the association of intensity threshold settings to surface fluorescence amount analysis.

Threshold A		
Intensity setting	r^2^	*p*-value
Threshold 1	0.91 (91%)	<0.0001
Threshold 2	0.89 (89%)	<0.0001
Threshold 3	0.85 (85%)	<0.0001

N = 83.

## 4 Discussion

In this study, a semi-quantitative scoring system was developed and further validated through image analysis to measure fluorescence. The visual scoring was based on the amount of fluorescence on the surface of the lymph node. Semi-quantitative scoring was assessed against two different image analysis thresholds to compare the stringency between the analysis settings. Data demonstrated that both thresholds had concordance against scores observed *ex vivo* and subjective positive/negative fluorescence status. However, data using Threshold A had almost perfect agreement with scoring and visual status assessment compared to subpar agreement of Threshold B. This finding resulted from the cut-off point used in Threshold A where Intensity set at 30–255 allowed pixels that are a dark blue fluorescent hue to be included in the measurement. In contrast, Threshold B did not include these dull or faint fluorescent values which illustrates the limitations of this threshold and the subsequent disuse for further analyses. The semi-quantitative scoring system is adapted from immunohistochemistry (IHC) and immunofluorescence principles, which is the gold standard performed at the microscopic level for tissue and cellular staining (35, 36). Due to prominent ICG visualization on the surface of surgical specimens, IHC scoring methods can be applied to create semi-quantitative information about the visible fluorescence.

The developed ICG scoring system had substantial intra and interobserver agreement. The lack of variability between the two observers shows that this assessment system can be done by different investigators and still yield the same score for a given sample. Variability in scores between observers occurred when fluorescence was faint and a dark blue hue, which can be due to ambient light, poor perfusion or obstructed visibility by fat tissue. When visual fluorescence status was compared between observers there was also perfect concordance, illustrating that there is very low bias and variance between observers in assessing fluorescence at a visual level. This concordance verifies that the observers have a similar understanding of what is deemed positive or negative fluorescence, making identifications of false positives or negatives negligible. When comparing individual observer scores to the Threshold A settings, Threshold A strongly agreed with scores from different observers. This observation further solidifies that when the gold standard uses parameters such as Threshold A, it does not eliminate potential lymph nodes that have dull fluorescence or nodes with a small amount of fluorescence. Finally, high fidelity between intraoperative and postoperative scoring shows the reproducibility of the scoring system *in situ*. This reproducibility can be helpful for clinicians and researchers who are evaluating fluorescence in real-time, without consultation from analysis programs. When Threshold A was compared to intraoperative scoring, there was strong concordance between the intraoperative scores and Threshold A. This concordance confirms that image analysis using software strongly agrees with scoring *in situ*. This illustrates the potential usage of a semi-quantitative scoring system to assess fluorescence when imaging systems fail to quantify fluorescence.

Normally, studies that use scoring systems for clinical assessment of surgical fluorescence resort to methods that are subjective and descriptive (24, 25, 37). Terms are often based on how visible the fluorescence is, for example “moderate fluorescence,” “hardly visible,” or “weak contrast” (25, 38). Despite these being clinically accessible forms of assessment, there are limitations to the reproducibility and standardization of subjective scoring methods. Subjective scoring with descriptor words heavily relies on the observer’s perception of the images and more than one observer is necessary to prevent observer bias and variability that could impinge on the reproducibility of the scoring system (24, 35). Even though the studies by Poellinger et al. (24) and Yokoyama et al. (25) established attempts to standardize the scoring system, they fail to justify the scores with a gold standard or quantification system, leaving room for further study (35).

The impact of threshold parameters on FI measurements was evaluated via the average FI of each lymph node image. Average fluorescence intensities between Threshold 1 vs. 2 and Threshold 1 vs. 3 produced significantly different values, demonstrating that the threshold settings, even a few units apart, influence the FI detected and, therefore, should be carefully chosen to minimize bias. Typically, during image analysis, users will choose thresholds based on a group of pictures and observe which threshold fits best (31). To evaluate the optimal threshold, for both intensity and amount of fluorescence, the pixel intensity histograms provided by the analysis software was used in this study to find a cumulative threshold that could be applied confidently to a batch of images (31). The overall threshold provided an estimate of the fraction of thresholding intervals to use, and which ones are statistically representative. Moreover, based on the pixel intensity histograms, three thresholds were further verified for significance through statistical tests that were then assessed for clinical research applicability.

To decide which threshold is optimal to quantify ICG fluorescence intensity, average FI values from each threshold were compared to fluorescence status based on visual observation and image analysis. Scores and the amount of fluorescence were transformed to positive (1) and negative (0) fluorescence status to fit the statistical model. Based on the results, the most practical and clinically applicable threshold parameter was Thresholds 2 and 3 where Intensity was set at 35–255 and 45–255, respectively. Threshold 1 was a significant predictor of fluorescence when tested against the visual status assessment, however it resulted in lower sensitivity and specificity compared to the other thresholds, resulting in false positives and negatives. Thresholds 1 and 2 were not found to be significant predictors of the gold standard fluorescence status test. However, this outcome is not indicative of the performance of these thresholds since the gold standard assessment is less inclusive and has higher detection of fluorescent pixels than the visual assessment. Therefore, this result does not reflect a clinician’s evaluation of surgical tissue specimens. Threshold 1 possessed more inclusivity and leniency that potentially measured non-fluorescent signals such as reflections of light from the exoscope and operating environment and background non-fluorescent tissue. Conversely, Thresholds 2 and 3 are optimal settings to predict fluorescence and measure FI based on their high sensitivity and specificity in detecting fluorescent nodes. As it is more clinically relevant when FI measurements include more false positives than omit them, the higher the specificity the more useful the test (39). Additionally, the statistical test produced an intensity cut-point value for future data to gauge at which intensity level a lymph node is deemed to have significant fluorescent intensity. Threshold 2 produced an intensity cut-point of 44.56 AU, while Threshold 3 produced a cut-point of 48 AU. Due to the strictness of image analysis in determining the status of fluorescence, a statistically significant cut-point based on this, such as 48 AU, potentially disregards dull fluorescence in lymph nodes, increasing the chances of false negatives. The cut-point of 44.56 AU limits false positives while maintaining fidelity, making it the simplest and clinically applicable predictor of true ICG FI in lymph nodes. Measuring FI via image analysis software is the gold standard for fluorescence microscopy. The rationale for comparing between FI image analysis protocol to visual fluorescence assessments would be to determine what is clinically relevant to the surgeon. Intensity measurements in image-guided surgery depend on what the surgeon can see *in situ*, therefore a visual status of fluorescence is a good indicator of sensitivity on the intensity measure.

Finally, the relationship between average FI and surface amount of fluorescence was evaluated and a strong association between average FI for all threshold settings and the amount of fluorescence on the surface of the node was found (Figure 2B). This implies a monotonically increasing relationship wherein a high amount of fluorescence measured on the lymph node correlates with high intensity FI values. This signifies that both average FI and surface amount of fluorescence influence the degree of fluorescence visualization, thus either can be chosen as a reporting measure when researching or collecting data in SLN mapping. However, FI measures are recommended since this is more translatable amongst different fluorescent imaging agents and imaging systems (21).

A strength of this study was the high degree of agreement and significance found in the results that allows the sample size to be sufficient. Additionally, there was an almost equal distribution of positive and negative lymph node samples (48 positive SLNs and 35 negative SLNs). The study also contained rigorous protocols to assess fluorescence. For example, the standardized nature of *ex vivo* imaging and the rigidity of the MetaMorph program caused background signals to be void. In addition, the protocol evaluated different threshold cut points that fine-tuned the analysis process for both FI and surface amount of fluorescence. Specifically, for FI the “Blue” channel was selected because it allows the MetaMorph program to focus intensity measures in the “Blue” channel of the RGB image, disregarding “Green” and “Red” intensity signals which do not correspond to the fluorescence in the image. A limitation in this study is the use of pseudo-colored images instead of raw fluorescence images since the VITOM II ICG does not provide raw images. Using pseudo-colored images prevents the analysis program from defining the lowest possible fluorescence signal and instead separates the lowest detectable fluorescence signal within the white light photographic image. This also limits the dynamic range of the signal between 0 and 255. It is recommended in the literature to use raw fluorescence images, if possible, to promote further precision and avoid color separation using hue (40). Another limitation of this study was the lack of automation to further the objectivity of the analysis process. An automated method for creating ROIs would be efficient and robust, however lymph nodes vary in size and shape which make it challenging to tailor specific macros for ROI creation. There are also no standardized methods for ROI selection available (21). The MetaMorph program allows for distinct ROI creation and minute ROI modifications to increase the accurate representation of the measured lymph node area. Additionally, due to standardized backgrounds and strict thresholding, ROIs adjacent to the lymph node area did not detect false background signals. Selection bias is avoided due to ROIs being a representative sample of the whole lymph node specimen (41). Another potential limitation is that residual bias may still exist in choosing thresholds, unlike an automated macro (42). Since our method is based on the SBR principle to quantify fluorescence in terms of amount and intensity, it was important to measure the background fluorescence that the threshold may detect (21). However, due to the standardized nature of our study, there was zero background fluorescence detected when the threshold was applied to every lymph node image, eliminating the need to use the SBR. This potentially decreases any human bias in threshold selection.

Our method for calculating the amount of fluorescence provides information about the amount of fluorescence on the surface of a lymph node that can be seen visually and through analysis. This information aids studies evaluating the uptake of fluorophores in tissue intraoperatively or *ex vivo*. The semi-quantitative assessment, in tandem with image analysis, allows a clinically accessible quantification system to classify lymph nodes during SLN mapping more objectively, straying away from the usual subjective terms (43, 44). Though this study uses whole specimen lymph nodes to evaluate our workflow, this can be extended as a general algorithm to apply to any whole tissue under interrogation that uses fluorescence. Additionally, the potential to implement more semi-quantitative assessments during surgery when not available through the imaging system may promote the advancement of the fluorescence-guided surgical technique to advanced stage trials, where there is a required criteria for determining positive fluorescence (43). Our study also describes a standardized method evaluating ICG FI and one that can easily be translatable to other fluorescent dyes. Currently, SPY-Q is able to quantify FI at the same scale as our method, where SPY-Q analyzes fluorescence imaging video sequences for FI in gray scale of 256 (AU) (45). Future research aims to compare our analysis method to systems, such as SPY-Q, that can quantify fluorescence. Imaging systems with quantification algorithms utilize comparable FI scales to those available in MetaMorph for lymph node images (AU of 255). This similarity implies that our system of measuring ICG fluorescence can be congruent to the quantification provided by commercially available imaging systems. More research is required to evaluate the application of our method in fluorescence-guided surgery using other systems, like VITOM II ICG, that lack built-in quantification.

## 5 Conclusion

In conclusion, we developed a standardized visual assessment and analytic system to quantify fluorescence by surface amount and intensity when imaging systems cannot provide this information during SLN mapping with fluorescence-guided surgeries (ICG and similar). The semi-quantitative scoring system had strong agreement with the analysis when Hue and Intesity were set at 165–180 and 30–255, respectively. In addition, Intensity set at 35–255 and 45–255 provide robust FI measures of fluorescent lymph nodes. The strong association between FI and the amount of fluorescence on a lymph node depicts the potential to use both measures interchangeably to quantify fluorescence. This assessment and analysis workflow provides the foundation for development of a system to quantify ICG visually and digitally, which may allow for standardized reporting of clinical research to improve comparability and consistency of results in SLN mapping of various cancers and tissue types utilizing ICG with different imaging systems. Future directions include the correlation of ICG distribution within lymph nodes to the localization of metastasis and morphological changes in lymph nodes as well as the impact on patient outcomes.

## Data availability statement

The datasets presented in this article are not readily available because NA. Requests to access the datasets should be directed to MO, moblak@uoguelph.ca.

## Ethics statement

Ethical approval was not required for the studies involving animals in accordance with the local legislation and institutional requirements because lymph nodes were collected as part of another study where approval/consent was collected or part of standard of care (negative controls). Written informed consent was not obtained from the owners for the participation of their animals in this study because this project centers around sample analysis once tissues have been excised.

## Author contributions

AR: Conceptualization, Data curation, Formal analysis, Investigation, Methodology, Validation, Writing – original draft, Writing – review & editing. KM: Conceptualization, Supervision, Writing – original draft, Writing – review & editing. CM: Writing – original draft, Writing – review & editing. JP: Supervision, Writing – original draft, Writing – review & editing. MO: Conceptualization, Data curation, Supervision, Writing – original draft, Writing – review & editing.
