# Supplementary material for: Validation of a semi-quantitative scoring system and workflow for analysis of fluorescence quantification in companion animals
Source: Front Vet Sci. 2024 Jul 31;11:1392504. doi: 10.3389/fvets.2024.1392504 (PMC11322124; doi:10.3389/fvets.2024.1392504)
Supplement: Supplementary file 1 [file Table_1.DOCX]

**Validation of a semi-quantitative scoring system and workflow for analysis of fluorescence quantification in companion animals**

**Supplemental Figures**

**Table S1** Patient and tumor characteristics of cases for SLN mapping with ICG

| **Case** | **Breed** | **Sex** | **Tumor type** | **Tumor location** |
| --- | --- | --- | --- | --- |
| 644764 | Mixed breed | MN | Melanoma | Oral (upper lip) |
| 645919 | Golden Retriever | MN | Mast cell tumor | Upper eyelid |
| 650537 | Boxer | M | Mast cell tumor | Scrotum |
| 656637 | Mixed breed | MN | Fibrous hyperplasia | Maxilla |
| 654045 | Mixed breed | MN | Ameloblastoma | Oral |
| 653832 | Golden Retriever | MN | Mast cell tumor | Left tricep |
| 651856 | Miniature Schnauzer | FS | Mast cell tumor | Left axilla |
| 635056 | Mixed breed | MN | Mast cell tumor | Left axilla |
| 619299 | Scottish Terrier | MN | Sarcoma | Left forelimb |
| 249770 | Dandie Dinmont Terrier | FS | Fibrosarcoma | Oral (cheek) |
| 654389 | Bull Mastiff | FS | Mast cell tumor | Left thigh |
| 654602 | Beagle | FS | Comedocarcinoma | Mammary gland |
| 660394 | Border Collie | MN | Ameloblastoma | Maxilla |
| 660291 | Labrador Retriever | FS | Osteosarcoma | Mandible |
| 660496 | Labrador Retriever | MN | Ameloblastic fibroma | Maxilla |
| 662249 | Mixed breed | MN | Osteosarcoma | Mandible |
| 662222 | Mixed breed | F | Melanoma | Lip |
| 655515 | Mixed breed | FS | Renal cell carcinoma^*^ | Left kidney |
| 655548 | Mixed breed | MN | Hematoma^*^ | Spleen |
| 657163 | Chihuahua | F | Adenocarcinoma^*^ | Thyroid gland |

^*^ These lymph nodes are ICG control cases.

Male (M); male neutered (MN); female (F); female spayed (FS)

**Table S2** Fluorescence image analysis measurements on negative controls

| Lymph node | Surface amount of fluorescence | | Background amount of fluorescence | Average FI | Background FI |
| --- | --- | --- | --- | --- | --- |
|  | Threshold A* | Threshold B |  |  |  |
| 1 | 0.045222 | 0 | 0 | 0 | 0 |
| 2 | 0.169558 | 0 | 0 | 0 | 0 |
| 3 | 0.000456 | 0 | 0 | 0 | 0 |
| 4 | 0 | 0 | 0 | 0 | 0 |
| 5 | 0.103299 | 0 | 0 | 0 | 0 |

*Threshold A detection under <1 is negligible and caused by light reflection.

**Table S3** Fluorescence image analysis measurements on negative controls

|  | Percentage agreement | Weighted kappa (95% CI) | Two-sided p-value |
| --- | --- | --- | --- |
| Score vs. Threshold B | 71.1% | 0.7984 (0.72 – 0.87) | <0.0001 |
| Visual fluorescence status vs. Threshold B | 92.8% | 0.8551 (0.74 – 0.97) | <0.0001 |
| Observer 1 vs. Threshold B | 72.2% | 0.7984 (0.72 – 0.87) | <0.0001 |
| Observer 2 vs. Threshold B | 71.1% | 0.8046 (73 – 0.88) | <0.0001 |
| Intraoperative vs. Threshold B | 71.1% | 0.7760 (0.69 – 0.86) | <0.0001 |
